# Development of novel and safer anti-breast cancer agents, SS1020 and SS5020, based on a fundamental carcinogenic research

**DOI:** 10.1186/s41021-019-0124-9

**Published:** 2019-03-28

**Authors:** Yoshinori Okamoto, Shinya Shibutani

**Affiliations:** 1 Faculty of Pharmacy, Meijo University, 150 Yagotoyama, Tempaku-ku, Nagoya, 468-8503 Japan; 2 Department of Pharmacological Sciences, State University of New York at Stony Brook, Stony Brook, 11794–8651 New York USA

**Keywords:** Anti-breast cancer agent, Tamoxifen, Endometrial cancer, DNA adduct formation, Metabolic activation, Estrogen receptor, Coactivator, Cancer therapy, Cancer chemoprevention

## Abstract

Tamoxifen (TAM) has been prescribed worldwide to patients with and women at high-risk of breast cancer. However, long-term use of TAM increases the incidence of endometrial cancer. The carcinogenic mechanisms of TAM have been extensively investigated. TAM is hydroxylated and sulfonated at α-carbon to form α-hydroxytamoxifen-*O*-sulfonate. This metabolite readily reacts with genomic DNA, particularly with 2′-deoxyguanosine, leading to DNA replication error. TAM also exerts estrogenic activity at endometrial tissue to induce endometrial hyperplasia. Therefore, our efforts focused on the development of novel and safer anti-estrogens to diminish carcinogenic potential of TAM based on chemical modifications. In this review, we describe a crucial idea of our drug design and introduce our compounds SS1020 and SS5020, possessing high effectiveness, and no genotoxic and estrogenic activities.

## Background

International Agency for Research on Cancer (IARC) reported in 2018 that new cases of all cancers in women was 8.6 million, among which 24.2% was of breast cancer [1]. Tamoxifen (TAM) has been prescribed for breast cancer prevention and treatment worldwide [2]. In Japan, TAM was launched in 1981 for treatment of estrogen receptor (ER)-positive breast cancer. Besides the beneficial effect of TAM, several epidemiological studies reported that TAM increased endometrial cancer incidence by 2–3 times as compared with placebo group [3–5]. Indeed, TAM-DNA adducts were detected in endometrial samples from TAM-treated patients [6–8]. Therefore, our research group has been developing safer anti-breast cancer agents since 2003 [9, 10]. Before introducing our compounds, we describe the mechanisms of carcinogenic effect induced by TAM and ideas for developing next-generation anti-breast cancer drugs.

## Carcinogenic mechanisms of tamoxifen and design of safer anti-breast cancer agents

Carcinogenic mechanisms of TAM have already been proposed to be its initiation and promotion effects. For the development of safer anti-estrogens, these two carcinogenic effects must be counteracted.

**Fig. 1:**
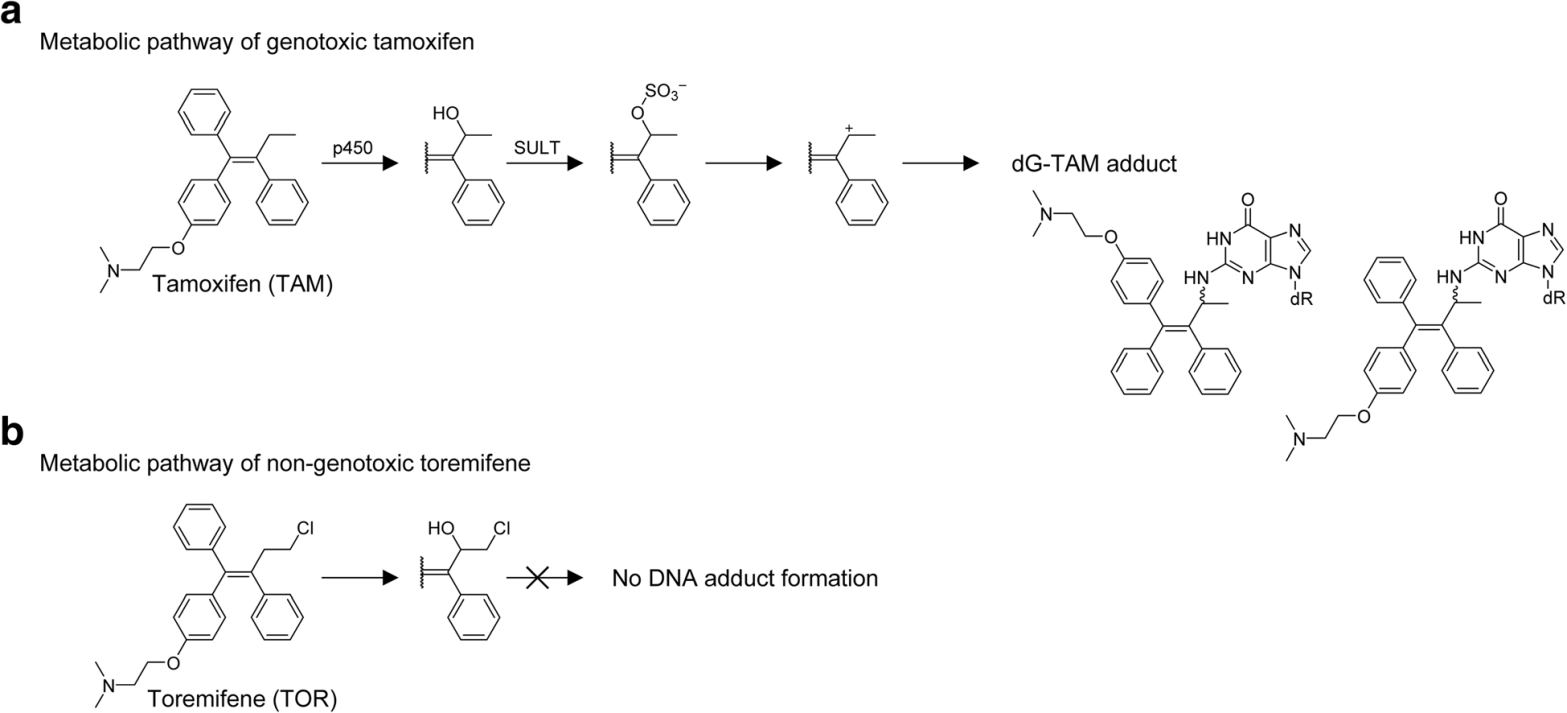
Genotoxic mechanisms of tamoxifen, not toremifene, via metabolic activation. TAM (**a**) proceeds four steps leading to genotoxicity which are 1) α-hydroxylation by cytochrome p450, 2) sulfonation by sulfotransferase (SULT), and 3) formation of carbocation to react with DNA, particularly with 2′-deoxyguanosine (dG) [11]. In contrast, TOR (**b**) is also hydroxylated at α-position. However, this metabolite is not a good substrate for sulfotransferases because of steric hindrance of bulky chloride atom [14]. As a result, toremifene doesn’t produce stable DNA adducts

Metabolic pathway of TAM leading to DNA modification was shown in Fig. 1. TAM proceeds hydroxylation at α-position, and then undergoes sulfonation by sulfotransferases to form α-hydroxytamoxifen-*O*-sulfonate, which is transformed to carbocation and readily reacts with 2′-deoxyguanosine (dG) in genomic DNA [11–13]. In contrast, toremifene (TOR), a chlorinated analog of TAM, also undergoes hydroxylation at α-position, whereas its α-hydroxylated metabolite is not a suitable substrate of sulfotransferases because of steric hindrance of bulky chloride atom [14, 15]. Therefore, DNA adducts were not detected in rat liver and human leukocyte samples [16–18]. The lack of genotoxicity in TOR is one of the critical findings for developing safer alternatives.

**Fig. 2:**
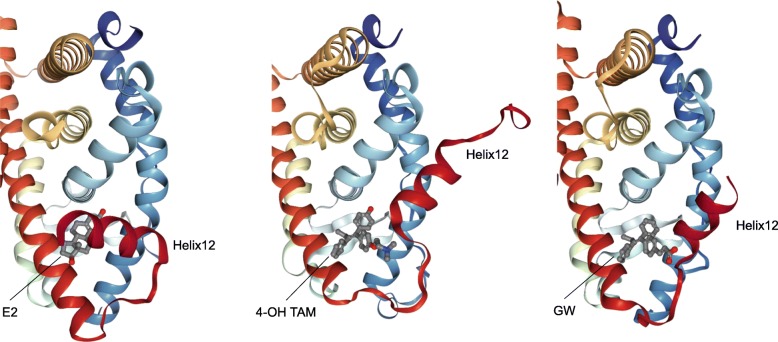
Overall structures of ligand-bound human estrogen receptor α ligand-binding domain complex. X-Ray crystal structures of human estrogen receptor α-ligand binding domain bound to E2 (left; PDB ID 1ERE [19]), 4-OH TAM (center; PDB ID 3ERT [21]) and GW (right; PDB ID 1R5K [26]) were illustrated using NGL software [20] on RCSB PDB website (http://www.rcsb.org/). These structures indicate the difference of helix (H) 12-relocation between three ligands. GW5638-induced H12 positioning increases surface hydrophobicity of ERα LBD, leading to ER instability

**Fig. 3:**
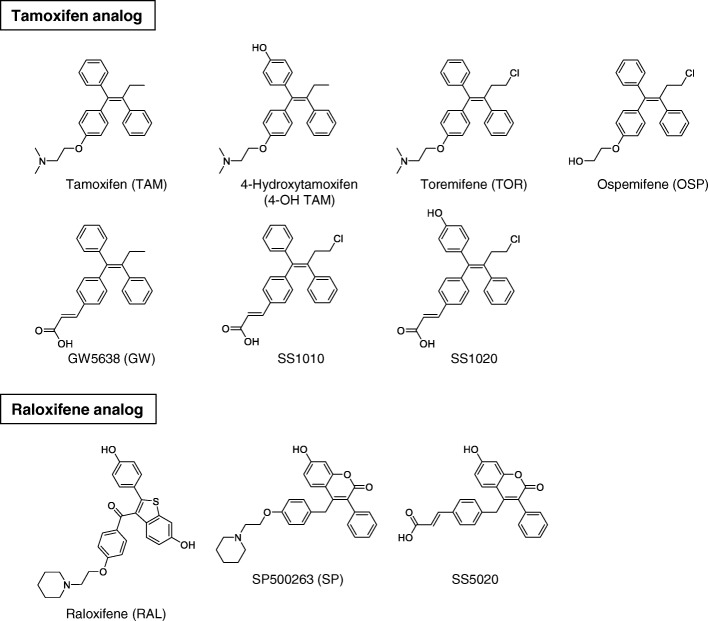
Chemical structures of SS1010, SS1020, SS5020, and related anti-estrogens

Regarding promotion activity, TAM is known as a selective estrogen receptor modulator (SERM), which exerts tissue-selective agonistic/antagonistic effects. TAM shows antagonistic effects on breast tissue, thereby suppresses the development of ER-positive breast cancer. However, TAM exerts agonistic effects on endometrial tissue, leading to endometrial cell proliferation. Unfortunately, non-genotoxic TOR also possesses agonistic activity in endometrial tissue in ovariectomized rats [9, 10]. Coactivator is a crucial determinant of ligand-dependent agonistic activity. X-ray crystal structure analysis revealed that 17β-estradiol (E2) induces appropriate relocation of the helix (H) 12 in ER-LBD (ligand-binding domain) [19], and allows ER to interact with specific coactivators, thereby leads to initiating transactivation (Fig. 2 [20]). In contrast, TAM induces H12 relocation different from that of E2, and suppresses recruitment of several coactivators, except SRC-1 (steroid receptor coactivator-1) [21]. SRC-1 expressing in endometrial cells can bind to TAM-bound ER complex; therefore TAM, probably TOR as well, shows an agonistic effect on endometrial tissue [22]. As shown in Fig. 3, there are many drug candidates for breast cancer treatment, among which GW5638 (GW) shows a unique anti-breast cancer property. GW is reported as effective against TAM-resistant breast cancer model with less estrogenic activity [23–25]. In hERα-LBD, acrylate side-chain of GW interacts with aspartate 351 residue in H12, leading to increased exposure of hydrophobic surface [26]. Since intracellular protein-degradation is increased depending on a surface hydrophobicity [27], acrylate side-chain would play an essential role in the decrease of intracellular ER protein by which GW shows differential anti-breast cancer spectrum as compared with TAM and TOR.

## Safer anti-breast cancer agents: SS1020 and SS5020

**Fig. 4:**
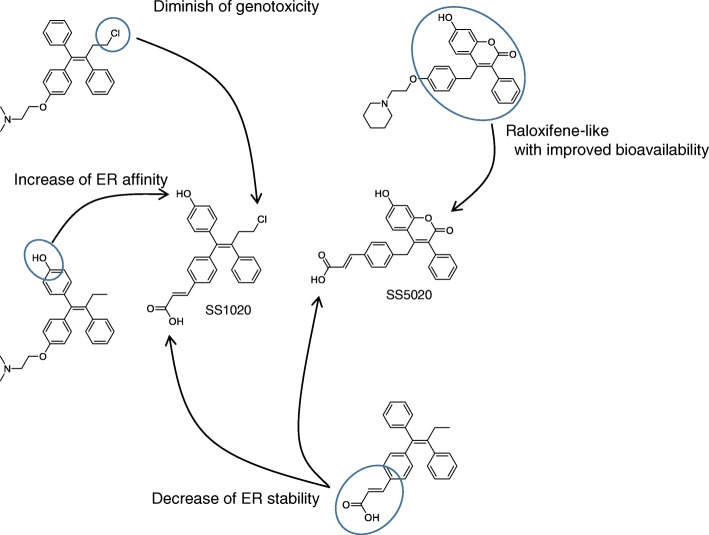
Basic ideas of structural design for potent and non-genotoxic anti-estrogens (SS1020 and SS5020)

To overcome the adverse effects of TAM, we designed SS1020 and SS5020 as safer alternatives (Fig. 4). Our compounds contain several essential structures as below. Chloride atom of SS1020 might diminish genotoxicity of TAM as shown in TOR. Also, 4-hydroxyl group of SS1020 promises to increase ER binding affinity as reported for 4-OH TAM [28]. Finally, the acrylate side-chain of SS1020 expects to diminish the endometrial activity of TAM with decreasing ER stability. Furthermore our ^32^P-postlabeling analysis showed that raloxifene (RAL) did not produce any DNA adduct in rat liver [17], the mimic of RAL structure is another choice for drug design. However, RAL, having two hydroxyl moieties, can be conjugated rapidly through phase II metabolism and excreted, making it difficult to achieve adequate bioavailability by oral administration [29]. Therefore, we synthesized SS5020 as a RAL analog with a slight structural modification to improve bioavailability [10].

**Fig. 5:**
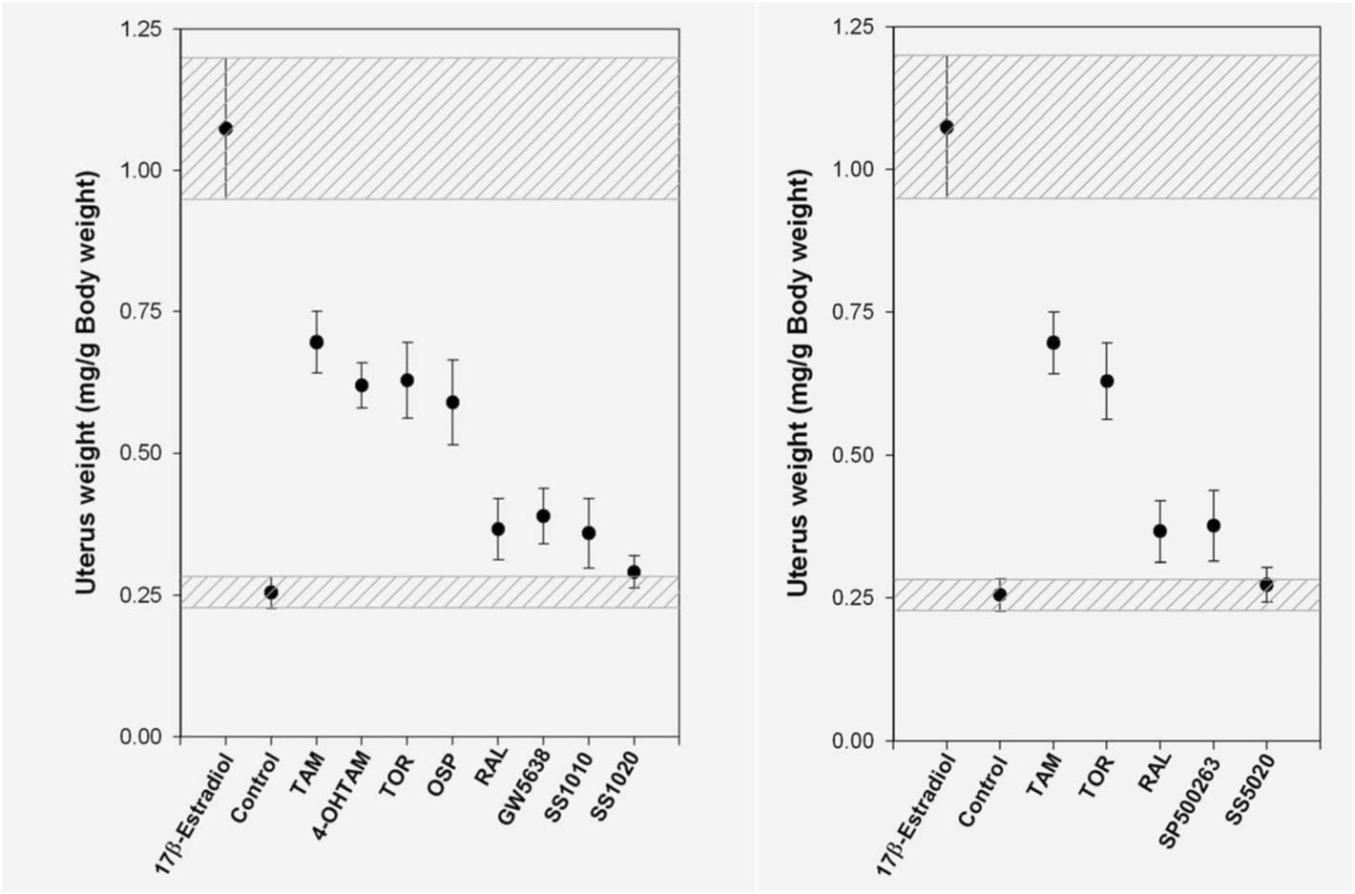
Uterotrophic effects of antiestrogens (left panel, SS1020 and its related compounds; right panel, SS5020 and its related compounds) in ovariectomized SD rats. Ovariectomized rats were subcutaneously treated with each test compound (0.3 μmol/rat/d of 17β-estradiol, or equivalent molar of test compound), and uterine wet weight was measured. TAM, 4-OH TAM, TOR, and OSP clearly increased uterine wet weight. RAL, GW, SP, and SS1010 exerted moderate uterotrophic effects. No detectable effects were observed in SS1020 [9] and SS5020 [10]

**Fig. 6:**
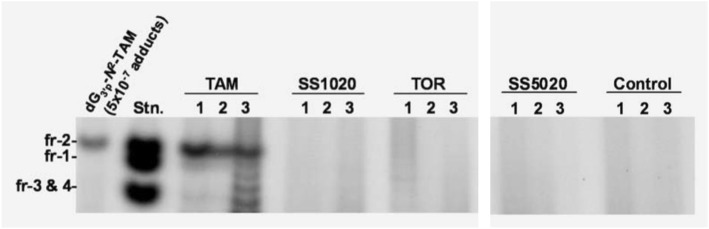
^32^P-postlabeling/PAGE analysis of SS1020, SS5020 and related anti-estrogens. Female SD rats were orally treated with test compound (20 mg/kg/d of TAM, or equivalent molar of test compound), and liver DNA was used for DNA adduct analysis. TAM treatment clearly formed dG-TAM adducts, whereas SS1020, SS5020 or TOR treatment didn’t observed any detectable DNA adducts [9, 10]

To confirm estrogenic activity of anti-estrogens, we carried out uterotrophic assay using ovariectomized rats in which rats were treated subcutaneously with test compounds for 3 d, and rat uteri were excised and weighted at 24 h after the final administration [9, 10]. TAM significantly increased uterine wet weight as shown in Fig. 5. 4-OH TAM, TOR, and ospemifene (OSP) also showed uterotrophic activity in this assay. RAL, SP500263 (SP) and SS1010 exerted moderate effects. As expected, SS1020 and SS5020 did not show any significant activity. Although the exact ER binding model of SS1020 and SS5020 remains to be unknown, our compounds might exhibit, at least in part, similar binding model, H12 relocation, and subsequent coactivator recruitment as seen in GW and SP. Therefore, we selected SS1020 and SS5020 for further determination of DNA adduct formation in rats (Fig. 6). Rats were orally treated with 20 mg of TAM or equimolar of test chemicals for 7 d, and rat liver DNA was analyzed using ^32^P-postlabeling/polyacrylamide gel electrophoresis assay [30]. In this assay, rat liver DNA was digested with nuclease P1 and micrococcal nuclease, and then DNA adduct was labeled with ^32^P using T4-polynucleotide kinase treatment in the presence of γ^32^P-ATP. Labeled DNA digests were separated on 30% polyacrylamide gel, and DNA adducts were observed as a single radioactive band. TAM treatment produced DNA adducts, especially dG-*N*^*2*^-TAM (fr-2), whereas no DNA adduct was detected in rat liver treated with TOR, SS1020 and SS5020. These results indicate that SS1020 and SS5020 have neither estrogenic activity nor genotoxicity in rats.

Anti-breast cancer potential of SS1020 and SS5020 was tested using two animal models which are dimethylbenz(*a*)anthracene-induced rat mammary tumor model and human breast cancer MCF-7 xenograft mouse model [9, 10]. In these animal experiments, SS1020 and SS5020 exhibit potent anti-breast cancer activity as compared with TAM, RAL, and GW. Although the mode of action should be further determined, SS1020 and SS5020 are considered to be safer alternatives for breast cancer therapy and prevention.

## Conclusions

In the last several decades, TAM has been prescribed for ER-positive breast cancer patients and women at high-risk for breast cancer. Epidemiological data indicated that TAM increased the incidence of endometrial cancer. This fact prompted us to develop a safer alternative, which our group succeeded to develop novel anti-breast cancer agents lacking genotoxicity and estrogenic activity. This project would provide an alternative option for women who hesitate to use TAM because of the concern about secondary endometrial cancer. However, most important, our project would pave the way for toxicologists to apply fundamental genotoxicity data for developing a novel drug design.

## Abbreviations

dG: 2′-Deoxyguanosine
E2: 17β-Estradiol
ER: Estrogen receptor
GW: GW5638
OSP: Ospemifene
RAL: Raloxifene
SERM: Selective estrogen receptor modulator
SP: SP500263
SRC-1: Steroid receptor coactivator-1
TAM: Tamoxifen
TOR: Toremifene
